# Nuclear relocation of Kss1 contributes to the specificity of the mating response

**DOI:** 10.1038/srep43636

**Published:** 2017-03-06

**Authors:** Serge Pelet

**Affiliations:** 1 Department of Fundamental Microbiology University of Lausanne Lausanne, Switzerland

## Abstract

Mitogen Activated Protein Kinases (MAPK) play a central role in transducing extra-cellular signals into defined biological responses. These enzymes, conserved in all eukaryotes, exert their function via the phosphorylation of numerous substrates located throughout the cell and by inducing a complex transcriptional program. The partitioning of their activity between the cytoplasm and the nucleus is thus central to their function. Budding yeast serves as a powerful system to understand the regulation of these fundamental biological phenomena. Under vegetative growth, the MAPK Kss1 is enriched in the nucleus of the cells. Stimulation with mating pheromone results in a rapid relocation of the protein in the cytoplasm. Activity of either Fus3 or Kss1 in the mating pathway is sufficient to drive this change in location by disassembling the complex formed between Kss1, Ste12 and Dig1. Artificial enrichment of the MAPK Kss1 in the nucleus in presence of mating pheromone alters the transcriptional response of the cells and induces a cell-cycle arrest in absence of Fus3 and Far1.

Mitogen Activated Protein Kinases (MAPK) are central nodes in the signaling network of eukaryotic cells. They are implicated in the transduction of extra-cellular information into defined cellular outputs. Diverse essential biological functions are associated with their activities such as growth, differentiation or stress response^1^,^2^,^3^. MAPKs have two main roles in the cell: the first one is to phosphorylate proteins, in order to modulate their activities; the second one is to induce a transcriptional program, which will alter the physiology of the cell in the long term^4^,^5^. MAPK substrates are located throughout the cell. They can be found at the plasma membrane, where the signal originally initiates from, in the cytoplasm or in the nucleus. The transcriptional response from the MAPKs requires a nuclear pool of the kinases, since it has been shown that these enzymes are closely associated with the transcriptional machinery and travel with the polymerase on the transcribed loci^6^,^7^.

Therefore, the partitioning of the MAPK between the cytoplasm and the nucleus is a key element of their function. It has been shown in mammalian cells that p38 and ERK can accumulate in the nucleus upon activation^8^,^9^. Similarly, in budding yeast, the p38 homolog Hog1 is strongly enriched in the nucleus upon hyper-osmotic stress^10^. In comparison, the nuclear to cytoplasmic partition of the MAPK Fus3 from the mating pathway does not change upon activation (Supplementary Figure 1a). Fus3 is enriched in the nucleus upon basal conditions and this enrichment remains constant after stimulation of the cells, despite a three-fold increase in global Fus3 concentration resulting from the activation of the transcriptional mating program^11^.

Interestingly, the group of Thorner has demonstrated that the nuclear function of Hog1 is dispensable for surviving hyper-osmotic challenge^12^. Indeed, a membrane associated Hog1 can phosphorylate its cytoplasmic substrates and thus enable adaptation without eliciting a transcriptional program. In contrast, a membrane bound or a nuclear restricted Fus3 are only partially functional since both cytoplasmic kinase activity and the nuclear transcriptional activity of Fus3 are necessary to mount a complete mating response^13^.

In parallel to Fus3, the MAPK Kss1 is also activated by mating pheromone^14^. This second kinase has been equally shown to be implicated in the filamentous growth and pseudohyphal growth in the Σ1287b diploids upon nutrient limitation^15^,^16^. Due to the number of shared components between the filamentous growth (FG) and the pheromone response (PR) pathways (Fig. 1a), many efforts have been dedicated to understand how signaling specificity is achieved between these two cascades. The signaling scaffold Ste5 has been identified as a key elements that favors the activation of the MAPK Fus3 upon mating pheromone stimulation^17^. In addition, the degradation of the FG specific transcription factor Tec1 by Fus3 prevents the induction of a filamentous transcriptional program in cells treated with mating pheromone^18^,^19^,^20^.

This paper characterizes the relocation of Kss1 out of the nucleus upon stimulation with mating pheromone. It demonstrates that this behavior is dependent on Fus3 or Kss1 activity. Artificial enrichment of Kss1 in the nucleus alters the cellular response in absence of Fus3, suggesting that this nuclear relocation contributes to the specificity of the signal transduction cascade.

## Results

### Signaling-dependent Nuclear Exit of Kss1

**Figure 1:**
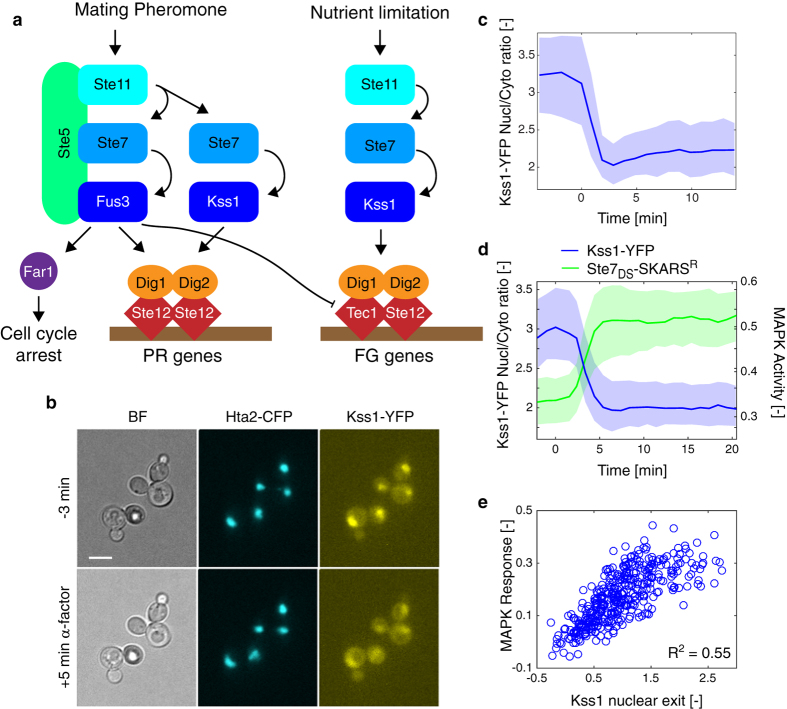
Kss1 is exported out of the nucleus upon mating pathway activation. (**a**) Schematic of the mating and filamentation growth pathways. (**b**) Cells bearing a histone tagged with CFP and Kss1-YFP were treated with mating pheromone (α-factor 1 μM) at time 0. Images, taken before (−3 min) and after (+5 min) the stimulus, demonstrate the relocation of Kss1 out of the nucleus. In all microscopy images the scale bar represents 5 μm (**c**). Time-lapse movies were automatically quantified and the ratio of Kss1 in the nucleus and in the cytoplasm is quantified for each cell (Nc = 300). For all similar graphs, the solid line is the median of all single cells. The shaded area represents the 25- and 75- percentiles of the population. Nc is the number of cells in the sample. (**d**) Comparison of the dynamics of Kss1 nuclear export and of MAPK activity in cells bearing a Kss1-YFP tag and a Ste7_DS_-SKARS^R^ (Nc = 806). (**e**) Correlation of Kss1 nuclear exit and MAPK activity in single cells 5 minutes after the stimulus.

To test whether Kss1 changes localization upon pathway activation, the endogenous copy of Kss1 was tagged with a YFP variant mCitrine in a strain bearing a histone tagged with CFP. Kss1 is enriched in the nucleus under normal growth conditions (Fig. 1b, Supplementary Movie 1). Interestingly, upon stimulation of the cells, Kss1 leaves the nucleus and redistributes into the cytoplasm. Using an automated image analysis platform, the average nuclear and cytoplasmic fluorescences of hundreds of cells were quantified in time-lapse movies^11^. The ratio between nuclear and cytoplasmic intensities is plotted in Fig. 1c. The sharp drop in this value after addition of pheromone at time zero represents the export of Kss1. As shown, the relocation from the nucleus is maximal 3 minutes after stimulus and remains constant after this time point.

The change in nuclear to cytoplasmic ratio arises mainly from a drop in the nuclear concentration of the MAPK. In parallel, a minor increase in cytoplasmic fluorescence can be monitored, which suggests that Kss1 relocates to the cytoplasm and is not degraded (Supplementary Figure 1b–d). Based on the relative size of the nucleus and cytoplasm^21^, it can be estimated that one third of the nuclear enrichment of Kss1 is lost. The residual enrichment of Kss1 in the nucleus is in agreement with the direct role played by the MAPK in the transcriptional process^7^,^22^.

In order to measure if kinase activity and Kss1 relocation are linked, a Kss1 allele tagged with mCitrine and a MAPK activity sensor (Ste7_DS_-SKARS^R^)^23^ were transformed in the same strain. This sensor consists in a fluorescent protein functionalized with a MAPK docking site from Ste7 (Ste7_DS_) and a nuclear localization sequence (NLS). Upon activation of the mating pathway, the MAPKs Fus3 and Kss1 specifically phosphorylate key residues close to the NLS. This modification alters the charge of the NLS, thereby decreasing the import of the construct in the nucleus and resulting in a net relocation of the fluorescent protein into the cytoplasm. The cytoplasm to nuclear ratio of the sensor can thus be used as a proxy for kinase activity. The exit of Kss1 out of the nucleus was synchronous with the activation of the MAPK cascade (Fig. 1d). At the single-cell level, the nuclear exit of Kss1 and the activity of the MAPK five minutes after stimulus are well correlated (Fig. 1e).

### MAP Kinase activity drives Kss1 nuclear exit

**Figure 2:**
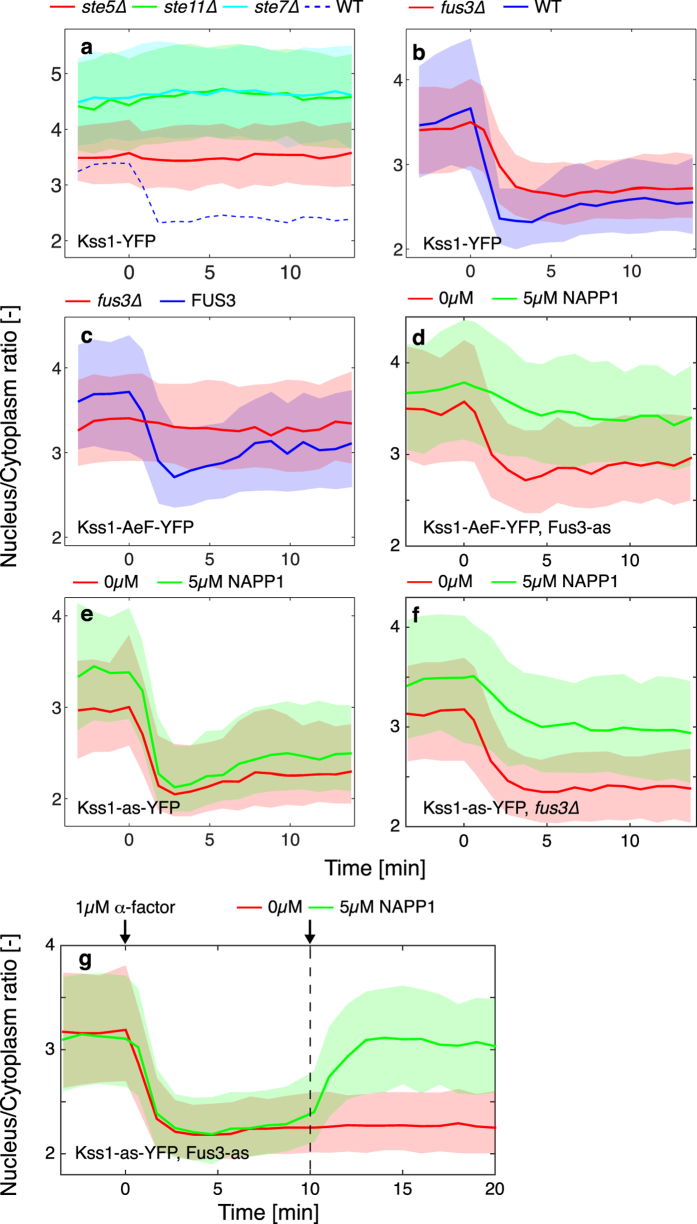
MAPK activity from either Fus3 or Kss1 drives the relocation of Kss1 out of the nucleus. (**a**) Nuclear to cytoplasmic relocation of Kss1 upon stimulation by mating pheromone at time 0 is absent in signaling dead mutants: *ste5∆* (red, Nc = 360) *ste11∆* (green, Nc = 906) and *ste7∆* (cyan, Nc = 374). The dashed blue line represents the response of the WT cells. (**b**) Comparison of the Kss1 nuclear relocation in WT (blue, Nc = 328) and *fus3∆* cells (red, Nc = 875). (**c**) Non-phosphorylatable mutant Kss1-AeF relocation is measured in presence (blue, Nc = 228) and absence (red, Nc = 359) of FUS3. (**d**) The non-phosphorylatable mutant Kss1-AeF is combined with an analog sensitive allele of FUS3 (Fus3-as). Addition of the inhibitor NAPP1 (green, Nc = 398) blocks the Fus3 kinase activity and strongly decreases the relocation of Kss1 compared to the control sample (red, Nc = 456). (**e**) Relocation of the Kss1-as allele in presence (green, Nc = 351) and absence (red, Nc = 253) of the NAPP1 inhibitor. (**f**) Relocation of the Kss1-as allele in presence (green, Nc = 591) and absence (red, Nc = 479) of the NAPP1 inhibitor in a *fus3∆* background. (**g**) Cells bearing both a Kss1-as-YFP and a Fus3-as allele were stimulated at time zero with 1 μM α-factor. Ten minutes later (dashed line), the NAPP1 (green, Nc = 607) was added to block kinase activity. Fast recovery of the nuclear Kss1 level is observed compared to a control sample (red, Nc = 701).

To verify more directly that the relocation of Kss1 is linked to signaling activity in the mating pathway, key elements of the cascade were deleted (Fig. 2a, Supplementary Movies 2 and 3). When the MAP kinase kinase kinase (MAP3K) Ste11, the MAP kinase kinase (MAP2K) Ste7 or the scaffold Ste5 are absent, Kss1 remains enriched in the nucleus. In *ste11∆* and *ste7∆* cells, the initial recruitment of Kss1 is higher than in the wild-type (WT) and *ste5∆*, probably because no basal MAPK activity is present in these mutants. Interestingly, in *fus3∆* cells, Kss1 exits the nucleus with slightly slower dynamics and to a lower extent than in WT cells (Fig. 2b, Supplementary Movie 4). Therefore, Kss1 relocation depends on signaling activity but is not specific to Fus3 activity. To test whether only the phosphorylated pool of Kss1 molecules can relocate, a mutant was constructed where two residues in the activation loop of the MAPK are mutated to non-phosphorylatable amino-acids (Kss1-AeF-YFP)^24^. This Kss1 allele displays a relocation phenotype, as long as Fus3 activity is present. Thus, combining a Kss1-AeF mutant with a *fus3∆* abrogates this response (Fig. 2c). To verify more specifically if the kinase activity of Fus3 is required for this relocation, an analog sensitive allele of Fus3 (Fus3-as)^25^ was introduced in this strain. Pre-treatment of the cells with the inhibitor NAPP1 strongly reduces the export of Kss1-AeF-YFP compared to a control sample treated with DMSO (Fig. 2d, Supplementary Movie 5 and 6).

From these data, it can be deduced that in the presence of a wild-type Kss1, Fus3 is not required for the relocation, while with a non-functional Kss1, Fus3 activity is required, underlying a putative redundant mechanism between the two MAPKs. Using an analog sensitive Kss1 (Kss1-as^26^, Supplementary Figure 2), it is possible to test whether Kss1 activity on its own can promote its relocation. Inhibiting Kss1 activity in *fus3∆* blocks the export of the MAPK, while it is not the case when Fus3 is present (Fig. 2e and f, Supplementary Movies 7 and 8). Taken together, these results demonstrate that either Fus3 or Kss1 activities are required to drive the nuclear exit of Kss1.

Finally, to test the reversibility of this nuclear depletion, cells combining the Fus3-as and the Kss1-as alleles were stimulated with pheromone and MAPK activity was blocked subsequently with NAPP1. Within less than five minutes after addition of the inhibitor, Kss1 recovers its original nuclear enrichment. This experiment clearly demonstrates the dynamic nature of the exchange between the nuclear and cytoplasmic pools of Kss1 (Fig. 2g, Supplementary Movie 9).

### Nuclear anchoring enriches Kss1 under normal growth conditions

**Figure 3:**
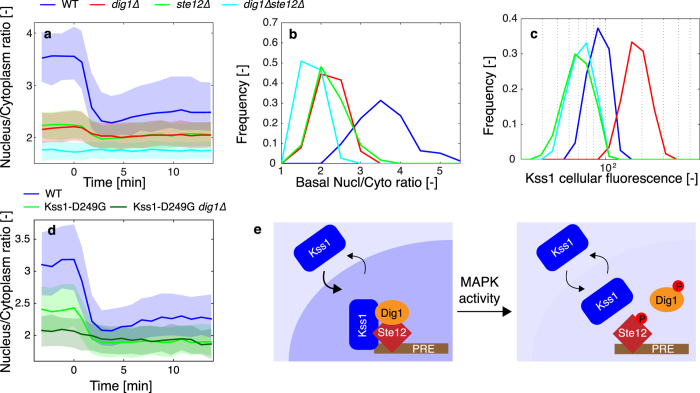
Nuclear enrichment of Kss1 under vegetative conditions is dependent on its association with Ste12 and Dig1. (**a**) Kss1-YFP nuclear relocation in WT (blue, Nc = 247), *dig1∆* (red, Nc = 440), *ste12∆* (green, Nc = 479) and *ste12∆dig1∆* (cyan, Nc = 431) upon stimulation of the cells with pheromone at time 0. (**b**,**c**). Histograms displaying the nuclear to cytoplasmic ratio (**b**) and the average cellular intensity (**c**) for the WT, *dig1∆* (red), *ste12∆* (green) and *ste12∆dig1∆* (cyan) before the α-factor stimulus. (**d**) Comparison of the nuclear relocation behavior of the MAPK insertion site mutant that disrupts the interaction between Kss1 and Ste12. WT: blue, Nc = 616, Kss1-D249G: light green, Nc = 439 and Kss1-D249G *dig1∆*: dark green, Nc = 218. (**e**) Schematic of the nuclear anchoring of Kss1. MAPK activity induced by pheromone disrupts the complex formed between Ste12, Dig1 and Kss1. The MAPK is then free to diffuse between the nucleus and the cytoplasm.

Since both Fus3 and Kss1 activities can drive the exit of Kss1 out of the nucleus, common nuclear substrates of the two MAPKs are likely to be involved in this process. Deletions of the transcription factor Ste12 and its repressor Dig1 decrease the enrichment of Kss1 under basal conditions and thus lower the difference between the inactive and active state of the pathway (Fig. 3a and b, Supplementary Movies 10, 11 and 12). Combined deletion of both proteins (*ste12∆dig1∆)* further reduces the basal enrichment of Kss1 in the nucleus and abolishes its relocation upon pheromone addition, suggesting an interaction between Kss1 and the Ste12-Dig1 complex, in agreement with previous studies from the Thorner lab^27^.

Deletions of Ste12 or Dig1 influence the expression level of Kss1 (Fig. 3c). In order to verify that the changes in expression levels are not responsible for the altered relocation phenotype, a constitutive promoter (p*ADH*) was placed upstream of the Kss1 coding sequence (Supplementary Figure 3a–c). Despite a modest overexpression of Kss1, the change in nuclear enrichment upon mating pathway activation can still be observed. Deletions of Ste12 and Dig1 in this strain decrease the nuclear enrichment of the MAPK under basal conditions and result in a weaker relocation of Kss1 in these mutants. Therefore, the reduced Kss1 enrichment observed in the *dig1∆* and *ste12∆* strains is not caused by a difference in expression levels, but rather due to the absence of factors anchoring Kss1 in the nucleus.

MAPKs are known to interact with their substrates via conserved domains such as the docking groove (common docking, CD)^28^,^29^,^30^ and the MAPK insertion site (MKI)^31^. A mutation located in this latter domain (Kss1-D249G) has been shown to abolish the interaction of Kss1 with Ste12^15^. This allele displays a lower basal enrichment in the nucleus and a weak relocation of Kss1 out of the nucleus upon pheromone treatment (Fig. 3d, Supplementary Movies 13 and 14). Combined with a deletion of Dig1, the basal enrichment of Kss1 is further decreased, resulting in an almost complete absence of relocation upon pheromone stimulus.

Docking groove mutants of Kss1 (Kss1-7m1 (D318A) and Kss1-7m3 (D318A, D321A)) suffer from a weak interaction with Dig1 as well as with Ste7 and Ste5^32^. As expected, both mutants show a decreased nuclear enrichment under basal conditions (Supplementary Figure 3d). Kss1-7m3, which is known to present a stronger phenotype compared to Kss1-7m1, displays a minimal enrichment of the MAPK in the nucleus prior to the stimulus and this localization remains unchanged throughout the time-lapse.

Kss1 has been shown to play a dual role in the FG pathway^15^,^16^. On the one hand, this enzyme functions as the MAPK in the cascade and it is therefore required to induce the transcriptional program. On the other hand, it stabilizes the Dig1-Ste12 complex under vegetative growth, thereby inhibiting the expression of filamentous genes in favorable growth conditions^27^. The dependence of the Kss1 relocation on the kinase activity of the two mating MAPKs, as well as the role played by Dig1 and Ste12 in its nuclear enrichment, are in line with these previous studies. In basal conditions, Kss1 associates with these two nuclear proteins via two independent interfaces: the CD and the MKI, leading to an enrichment of the MAPK in the nucleus. Phosphorylation of Dig1 and/or Ste12 upon pheromone stimulation breaks down this complex, allowing the transcription of the mating genes^33^,^34^ and a change in the partitioning of Kss1 between the nucleus and the cytoplasm (Fig. 3e).

### Artificial recruitment of Kss1 in the nucleus

**Figure 4:**
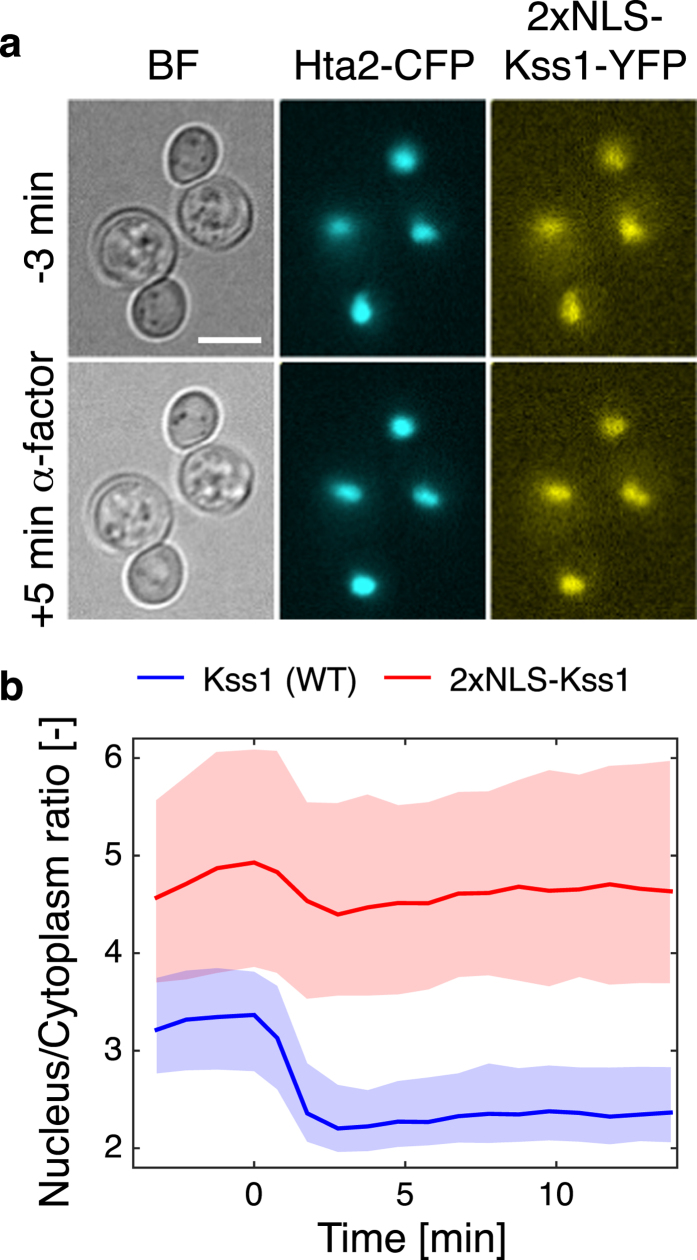
Characterization of a nuclear enriched allele of Kss1. (**a**) A nuclear enriched Kss1 allele is generated by the addition of two nuclear localization sequences upstream of the coding sequence of the proteins. The 2xNLS-Kss1-YFP is visualized by microscopy in cells bearing a histone tag CFP before (−3 min) and after (+5 min) α-factor addition at time 0. (**b**) Nuclear to cytoplasmic ratio of a WT and 2xNLS-Kss1 quantified during a time-lapse movie with addition of pheromone. WT: blue, Nc = 334; 2xNLS-Kss1: red, Nc = 677.

Activation of the mating pathway results in three well-characterized cellular outcomes: formation of a mating projection (shmoo), induction of a specific transcriptional program and arrest of the cell-cycle in G1. In order to test if the nuclear exit of Kss1 plays a role in the physiology of the response to mating pheromone, a double nuclear localization sequence (2xNLS) was added upstream of the MAPK coding sequence. As expected, the construct is more nuclear than a wild-type MAPK (Fig. 4a and b, Supplementary Movie 15). Upon addition of pheromone, this protein retains a high nuclear enrichment level due to the presence of the two NLSs. It has to be noted that this constitutive nuclear accumulation leads to a decrease in the overall fluorescence intensity of this strain, suggesting that this allele is expressed at a lower level or is less stable (Supplementary Figure 4a–e). However, due to the higher expression of Kss1 in absence of Fus3, the 2xNLS-Kss1 in *fus3∆* reaches similar nuclear concentrations as the WT kinase in WT background.

**Figure 5:**
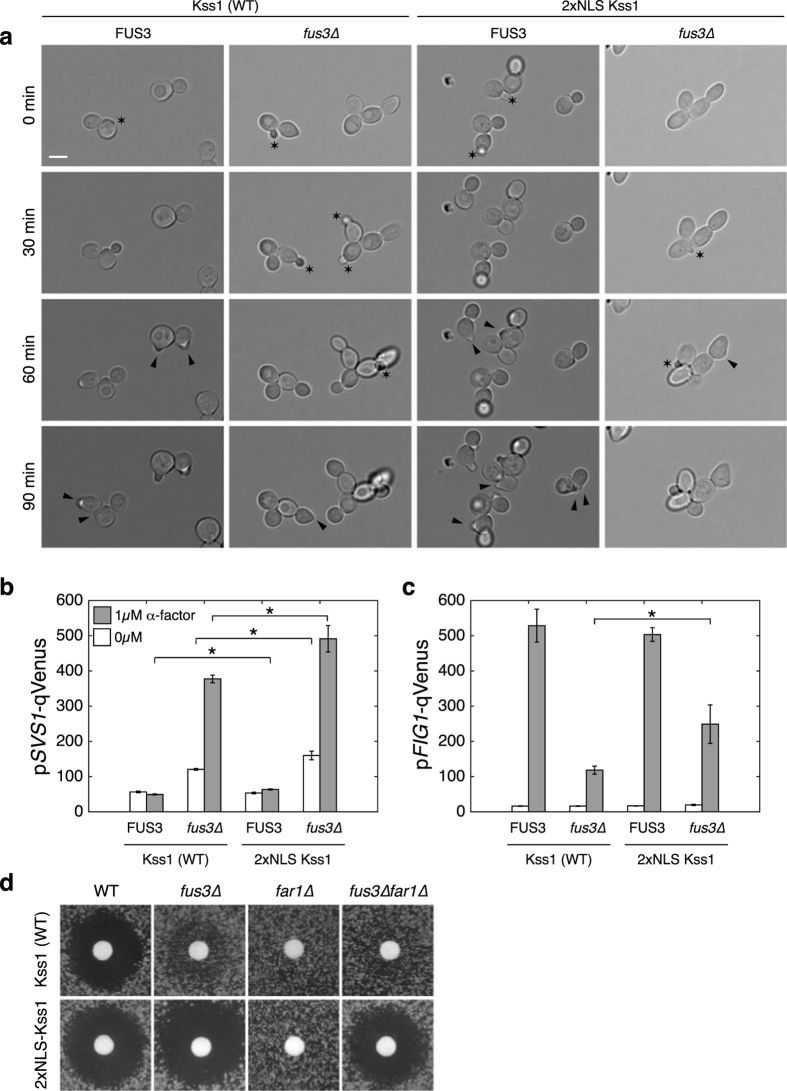
Nuclear accumulation of Kss1 in absence of Fus3 perturbs the transcription profile of the cells and leads to cell cycle arrest. (**a**) Morphologies of cells stimulated with pheromone at time 0 bearing either the WT or 2xNLS-Kss1 allele in presence or absence of Fus3. Black arrowheads indicate shmoo emergence. Black stars highlight emerging buds. (**b** and **c**) Quantification of the induction of a FG (p*SVS1*, **b**) and a mating (p*FIG1*, **c**) specific expression reporters. Cells were stimulated with α-factor for 1.5 hr before quantification of their fluorescence by microscopy. Each measurement represents the mean of three biological replicates, where on average 3000 single cells were quantified. The error bar is the standard deviation of these three replicates. The asterisks indicate statistically significant difference (t-test, p-value < 0.05) between Kss1 and 2xNLS-Kss1 samples. The measurements between 0 and 1 μM α-factor are all statistically significant. (**d**) Halo assays where 10 μl of α-factor 600 μM were dropped on a filter disk on a plate where yeasts of various backgrounds were plated. Note the striking differences between the Kss1 and the 2xNLS-Kss1 in *fus3∆* and *fus3∆far1∆* backgrounds.

Sixty to ninety minutes after stimulation with a saturating concentration of pheromone (1 μM), wild-type cells form mating projections (Fig. 5a, Supplementary Movies 16 to 19). In absence of Fus3, the cell-cycle arrest induced by a specific phosphorylation of Far1 by Fus3 cannot take place^35^,^36^, thus cells continue to bud and very few shmoos can be observed. The presence of the 2xNLS-Kss1 allele does not appreciably alter this situation. The *fus3∆* cells keep dividing, while the ones that possess a copy of FUS3 accumulate in G1 and form mating projections. One difference that can be observed is the tendency of 2xNLS-Kss1 *fus3∆* cells to have an elongated morphology (Supplementary Figure 4f), which is reminiscent of a filamentous growth phenotype^37^.

This altered shape prompted us to verify the relative level of filamentous versus mating gene expression in these four different backgrounds. In order to achieve this, fluorescent expression reporters based on the mating specific promoter FIG1^38^ or the Tec1-dependant promoter SVS1^39^ were introduced to quantify the relative induction of these two transcriptional programs (Fig. 5b and c). The fluorescence of log-phase or α-factor treated cells was quantified by microscopy. FUS3 positive cells displayed a strong and specific induction of the FIG1 promoter in presence of pheromone, independently of the presence of the 2xNLS-Kss1 allele. The SVS1 promoter is slightly repressed upon α-factor stimulation in wild-type cells, but this repression is absent in 2xNLS-Kss1 cells, leading to a small but statistically significant induction of p*SVS1*.

In *fus3∆* cells bearing the WT allele of Kss1, p*FIG1* induction is strongly decreased. In parallel, p*SVS1* is derepressed in basal conditions and its expression level increases three-fold when cells are treated with pheromone. These changes denote a shift of the transcriptional program towards a filamentous-like response. This can be explained by the lack of Fus3-dependent degradation of Tec1, which inhibits the production of filamentous genes upon α-factor treatment^18^,^19^,^20^. When combining the *fus3∆* with the 2xNLS-Kss1, the specificity of the cascade seems to be perturbed, since the nuclear Kss1 drives a strong induction of both mating and filamentation expression programs.

As mentioned above, another hallmark of the mating pathway activation is the cell-cycle arrest promoted by the phosphorylation of Far1. This arrest can be visualized by a halo assay where the addition of pheromone on a filter disk placed on a lawn of cells blocks the proliferation of the cells in the vicinity of the filter (Fig. 5d). This arrest is dependent on the presence of Fus3 or Far1, since cells lacking either of these proteins can proliferate in presence of the α-factor. Strikingly, cells bearing the 2xNLS-Kss1 construct in *fus3∆* background display a lack of growth in the region surrounding the paper disk. Even more surprising, this growth arrest does not require the Far1 protein, since the double mutant *fus3∆far1∆* also displays a halo.

Taken together, these results reveal that the artificial nuclear enrichment of Kss1 in absence of Fus3 alters the morphology of the cells under vegetative growth conditions, induces simultaneously the mating and FG transcriptional program upon pheromone treatment and produces a cell-cycle arrest after few rounds of divisions (Supplementary Figure 5). Therefore, the relocation of Kss1 in wild-type cells is likely to contribute to the specificity of the signal transduction performed by Fus3 and Kss1 in the mating and filamentous growth programs.

## Discussion

Fus3 and Kss1 orchestrate the response to mating and filamentous growth. The interplay between these two kinases is tightly regulated such that a specific signaling outcome can be established depending on the stimuli encountered by the cells. The temporal and spatial regulation of the activity of these two kinases has to be tightly controlled in order to deliver an appropriate response. The binding of Kss1 to Ste12 and Dig1 under normal growth conditions anchors the MAPK in the nucleus of the cells. Previous studies^15^,^16^ have shown that these interactions contribute to the repression of the FG transcriptional program.

Upon activation of the mating pathway, the Kss1-Dig1-Ste12 complex is disrupted, leading to a relocation of Kss1 into the cytoplasm. This relocation event is linked to the kinase activity of either Fus3 or Kss1, which can disrupt the Dig1 repression on Ste12 by phosphorylation. Dig2 probably plays a similar role as Dig1 in this process. However, under the tested experimental conditions, it was observed that Dig2 is expressed to a lower level than Dig1 and therefore displays a much weaker phenotype. The extent of nuclear Kss1 in basal conditions varies in different backgrounds. In *ste11∆* and *ste7∆* cells, where neither Fus3 nor Kss1 can be phosphorylated, Kss1 is more enriched in the nucleus than in *ste5∆* cells, where signal transduction is also impaired but phosphorylation of Kss1 remains possible.

Using two NLSs, the spatial regulation of Kss1 was perturbed by artificially enriching this protein in the nucleus. The regulation of the complex between Kss1, Ste12 and Dig1 is probably not affected by this change in Kss1 localization. However, when exogenous pheromone is added, Kss1 remains enriched in the nucleus. In presence of Fus3, a small induction of the p*SVS1*-qV reporter can be noticed, probably due to a stabilization of Tec1 by the 2xNLS-Kss1. When Fus3 is absent, a strong induction of both mating and filamentous growth expression reporters is observed with the 2xNLS-Kss1 allele, denoting a lack of specificity of the response.

The most surprising finding is that these cells display a growth arrest in a halo assay test in *fus3∆* and *fus3∆far1∆* cells. Far1 orchestrates the cell-cycle arrest of cells treated with pheromone. It is directly phosphorylated by Fus3 (and not by Kss1), which leads to its stabilization and thus prevents the cyclins to promote a new entry in the cell cycle. In the 2xNLS-Kss1 allele, a Far1-independent arrest is observed. Other instances of Far1-independent arrests have been found in the literature^40^,^41^,^42^,^43^. One explanation for an arrest in G1 is the role of Fus3 and Kss1 in repressing the transcription of G1/S cyclin genes (CLN1, CLN2, CLB5)^40^. In wild-type cells, Fus3 takes a major role in this process and thus in *fus3∆* only a mild phenotype can be observed (note the faint halo formed in Kss1-WT, *fus3∆* cells in Fig. 5d). However, the retention of 2xNLS-Kss1 in the nucleus promotes this arrest by altering the transcriptional program induced in these cells. Long time-lapse measurements of this phenomenon revealed that cells could undergo a few divisions before arresting in an unbudded state (Supplementary Figure 5). These time-lapse movies are in agreement with a mechanism where cells fail to enter a new cell-cycle due to the lack of transcription of the appropriate cyclins.

To conclude, nuclear anchoring of Kss1 via Ste12 and Dig1 participates in the repression of the filamentous genes under normal growth conditions. Eviction of the MAPK upon mating pheromone stimulation therefore contributes to the specificity of the mating response. In absence of Fus3, a nuclear enriched Kss1 will promote a gene expression program implicating both filamentation and mating genes, which compromises the growth of the cells.

## Methods

### Yeast Strains and plasmids

A list of strains and plasmids used in this study can be found in Supplementary Tables 1 and 2. All strains are of W303 background. Gene disruptions and gene tagging were performed by standard Li-Acetate transformation procedure using pFA6a-based vectors amplified by PCR^44^,^45^. The expression reporters pFIG1-qVenus and pSVS1-qVenus were cloned in pRS305 backbone and integrated in the LEU2 locus by digestion with ClaI^38^,^46^. The MAPK activity reporter is cloned in a pSIVu vector and integrated in the URA3 locus^47^.

To generate the various Kss1 alleles, the full sequence of Kss1-mCitrine including 940 bp of the promoter along with its CaURA3 tagging cassette was amplified from genomic DNA and cloned in a pRS313 backbone between XhoI and SacI. Sub-cloning or point mutations using the Q5 site-directed mutagenesis kit (E0554S, NEB) were performed to obtain the desired mutants, which were fully verified by sequencing. The plasmids were digested with SacII and KpnI and the cassettes were transformed in a *kss1∆* strain bearing a Hta2 tagged with CFP. Transformants were screened by microscopy to verify the proper integration of the fluorescent Kss1 construct.

### Sample preparation

Yeast strains were grown overnight to saturation in synthetic medium (CYN6202, DCS0031, ForMedium). The cultures were diluted in the morning to OD 0.025 and grown for at least 4 hours. Prior to imaging, cells were diluted to OD 0.03 and briefly sonicated to break clusters of cells. Two hundred microliters of culture were transferred into a 96-well plate (MGB096-1-2LG, Matrical Bioscience), with wells coated with Concanavalin A (0.5 mg/ml, C2010-250MG, Sigma-Aldrich). Prior to imaging, cells settled to the bottom of the plate for 30 min in the microscope incubation chamber set at 30 °C. Two or three frames after the start of the acquisition, 100 μl of a 3 μM α-factor solution were added to each well to stimulate the cells, resulting in a final concentration of 1 μM. Analog sensitive alleles of Fus3 and Kss1 were inhibited by addition of 5 μM NAPP1 (A603004, Toronto Research Chemicals) twenty to thirty minutes before stimulation with pheromone. Control experiments were performed by treating the cells with a similar amount of DMSO (0.8 μl in 1ml SD-full). For the quantification of the p*FIG1* and p*SVS1* promoter induction, cells were stimulated with α-factor in microtubes and grown for one hour and a half in a shaking incubator at 30 °C. At this time point, protein expression was blocked with cycloheximide (0.1 mg/ml) and the synthesized fluorescent proteins were allowed to fully mature for one additional hour before measurement under the microscope.

### Microscopy

Detailed protocols for microscopy measurements and data analysis have been published previously^11^,^48^. Briefly, images were acquired on a fully automated inverted epi-fluorescence microscope (Ti-Eclipse, Nikon) controlled by micro-manager^49^ with a 40X oil objective and appropriate excitation and emission filters. The excitation is provided by a solid-state light source (SpectraX, Lumencor). The images were recorded with a sCMOS camera (Flash4.0, Hamamatsu). A motorized XY-stage allowed recording multiple fields of view. At each XY position, multiple fluorescent and brightfield images were acquired for every time point. Time-lapse movies were analyzed with the YeastQuant platform^11^. The nuclei of the cell were segmented by thresholding of the CFP images. The contour of the cell around each nucleus was detected using two brightfield images, allowing to define a cytoplasmic object around each nucleus. The ratio of the average nuclear over cytoplasmic fluorescent intensity is calculated. Only cells tracked from the beginning to the end of the movie were taken into consideration for the analysis. At least three biological replicates for each mutant were measured and one representative data set is displayed in the Figures.

## Additional Information

**How to cite this article:** Pelet, S. Nuclear relocation of Kss1 contributes to the specificity of the mating response. *Sci. Rep.* **7**, 43636; doi: 10.1038/srep43636 (2017).

**Publisher's note:** Springer Nature remains neutral with regard to jurisdictional claims in published maps and institutional affiliations.

## Supplementary Material

Supplementary Information

Supplementary Movie 1

Supplementary Movie 2

Supplementary Movie 3

Supplementary Movie 4

Supplementary Movie 5

Supplementary Movie 6

Supplementary Movie 7

Supplementary Movie 8

Supplementary Movie 9

Supplementary Movie 10

Supplementary Movie 11

Supplementary Movie 12

Supplementary Movie 13

Supplementary Movie 14

Supplementary Movie 15

Supplementary Movie 16

Supplementary Movie 17

Supplementary Movie 18

Supplementary Movie 19
