# Supplementary material for: Nuclear relocation of Kss1 contributes to the specificity of the mating response
Source: Sci Rep. 2017 Mar 6;7:43636. doi: 10.1038/srep43636 (PMC5337980; doi:10.1038/srep43636)
Supplement: Supplementary Information [file srep43636-s1.pdf]

Supplementary Information to:

Nuclear relocation of Kss1 contributes to the specificity of the mating response

Serge Pelet

Department of Fundamental Microbiology
University of Lausanne
Lausanne, Switzerland

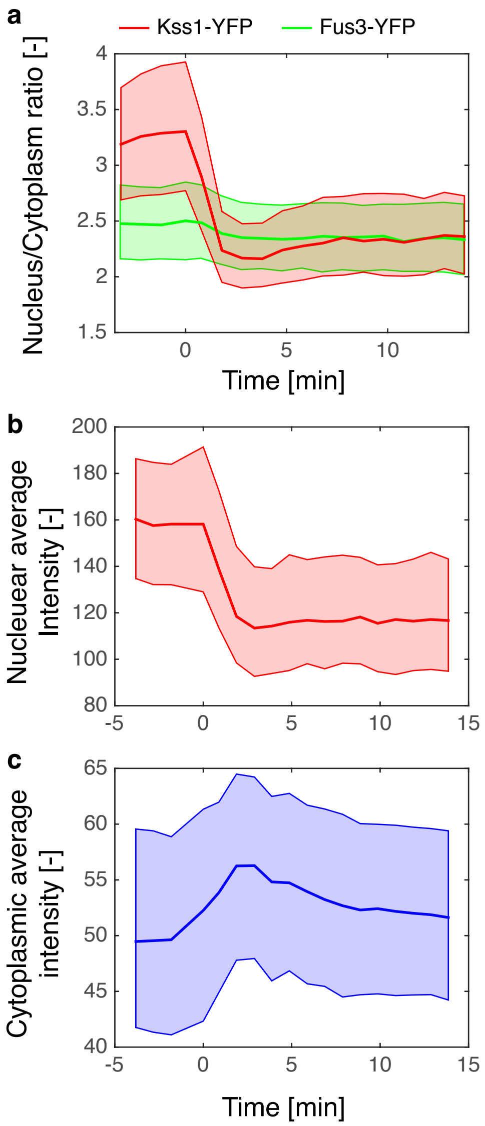

d

Nuclear volume: 2.9μm^3
Cell Volume: 42μm^3
Ratio: 7%

Thus the average concentration of Kss1 in the cell is 0.217 μM
Before stimulus, based on an N/C ratio of 3:
[N] = 0.571 μM [C] =0.190 μM

After stimulus, based on an N/C ratio of 2:
[N] = 0.405 μM [C] =0.202 μM

Supplementary Figure 1. Quantification of Kss1 nuclear relocation and comparison with Fus3
a. Comparison of the relocation behavior of Kss1-YFP (red, Nc = 724) and Fus3-YFP(green, Nc = 1010). In contradiction to previous studies from the Stone's Lab (Blackwell *et al.* Molecular and Cellular Biology, 2003), no nuclear accumulation of Fus3 at early times can be detected in our experiments. **b.** and **c.** The nuclear (b) and cytoplasmic (c) average fluorescent intensity for the data plotted in Figure 1c. Mean nuclear intensity levels of Kss1 and 2xNLS-Kss1 in WT and *fus3*Δ cells as function of time after stimulus. **d.** Simple calculation of the nuclear and cytoplasmic concentrations of Kss1 before and after stimulus based on the cellular and nuclear volume measured in Jorgensen *et al.* Molecular biology of the cell, 2007 and the number of Kss1 molecules per cell from (Ghaemmaghami *et al.* Nature, 2003).

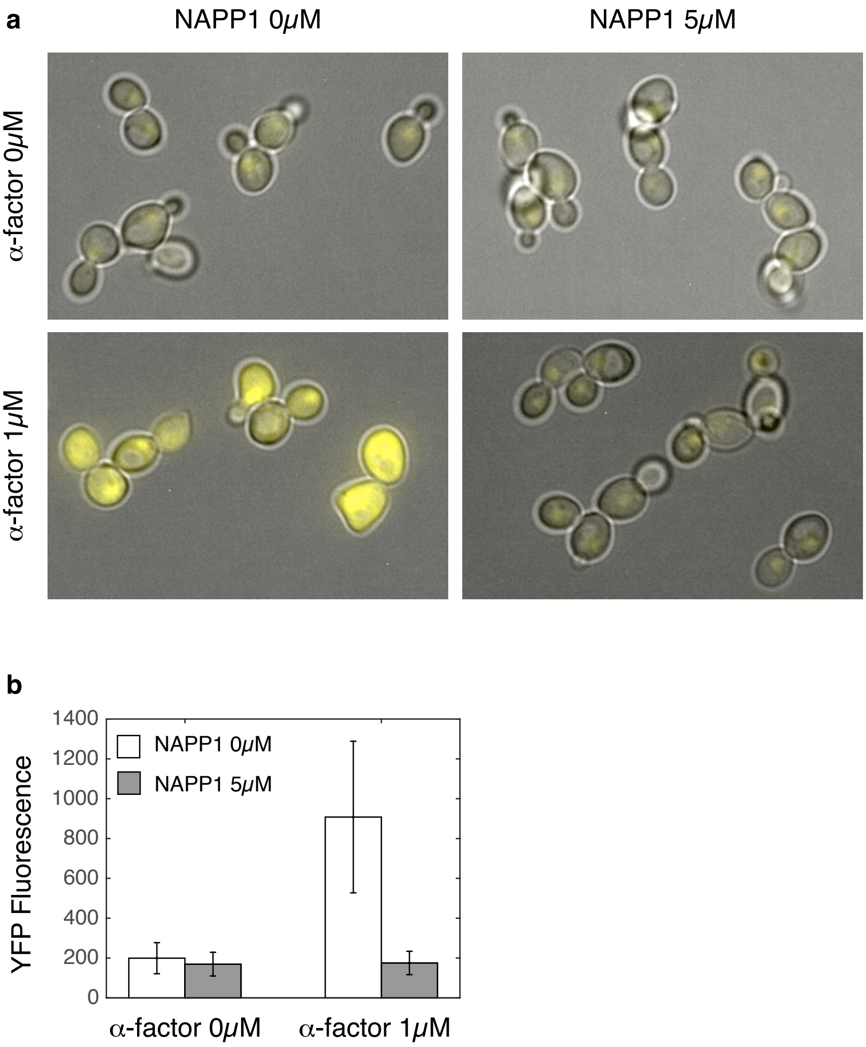

Supplementary Figure 2. Verification of the inhibition of Kss1-as by NAPP1

a. Cells bearing the Kss1-as-YFP allele in a *fus3*Δ background were transformed with a fluorescent expression reporter p*FIG1*-qVenus. Cells were treated for 10 min with NAPP1 5mM or a DMSO control prior to stimulation with α-factor or a SD-full control. Addition of NAPP1 clearly inhibits the apparition of fluorescence and the formation of mating projections. **b.** Quantification of the average cellular YFP fluorescence in the four different conditions. The mean and the standard deviation of the population (Nc >800) are plotted.

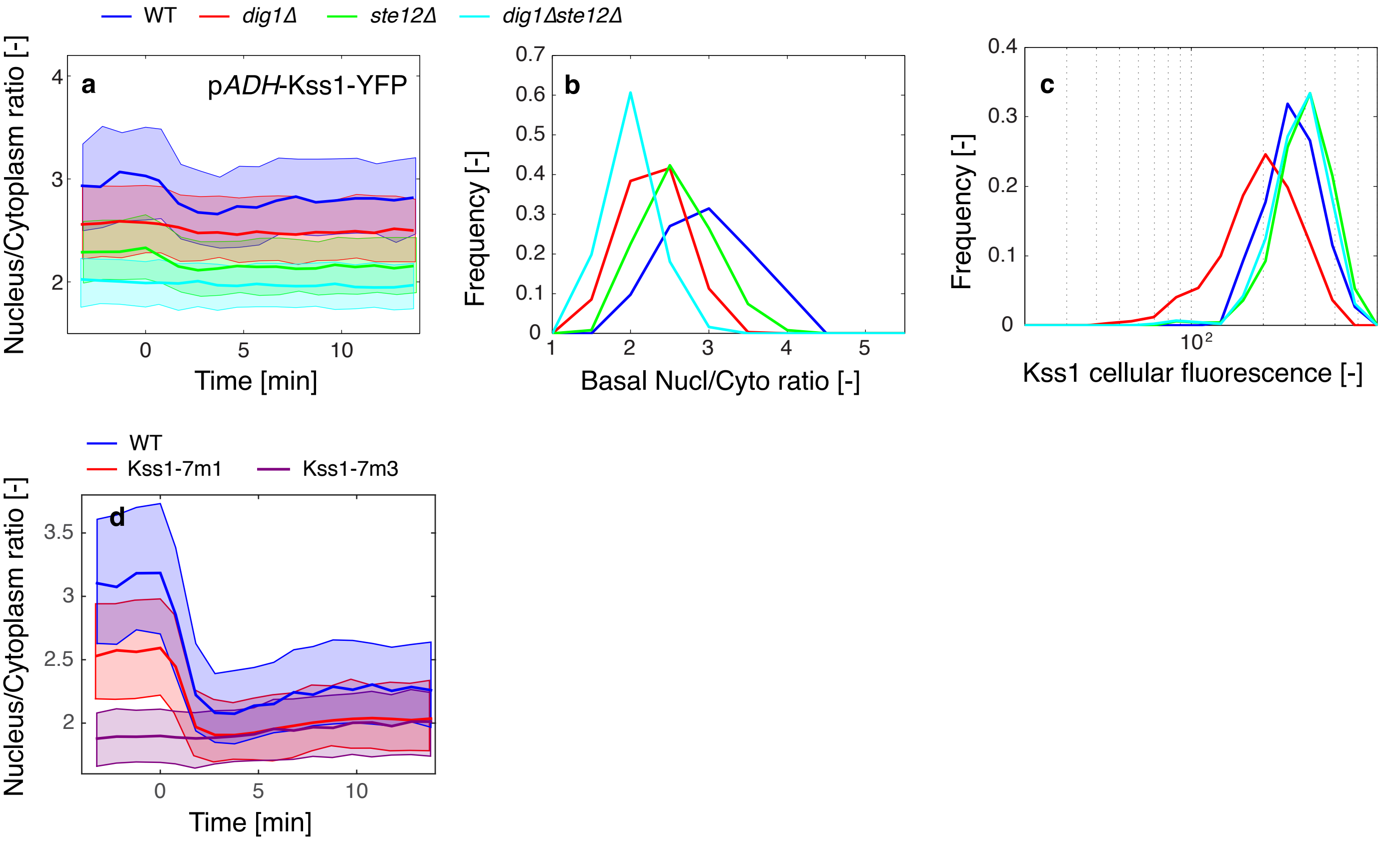

Supplementary Figure 3. Role of Dig1 and Ste12 in Kss1 nuclear relocation.
a. p*ADH*-Kss1-YFP nuclear relocation in WT (blue), *ste12*Δ (green), *dig1*Δ (red) and *ste12*Δ*dig1*Δ (cyan) upon stimulation of the cells with pheromone. **b.** and **c.** Histograms displaying the nuclear to cytoplasmic ratio (b) and the average cellular intensity (c) for the WT, *ste12*Δ(green), *dig1*Δ(red) and *ste12*Δ*dig1*Δ (cyan) before the α-factor stimulus. Despite similar expression levels of the consitutively expressed p*ADH*-Kss1-YFP construct, the relocation of Kss1 is altered similarly to WT cells in the *ste12*Δ*dig1*Δ **d.** Comparison of the nuclear relocation behavior for different alleles of Kss1 that disrupt the MAPK docking groove implicated in the interaction with Dig1 (7m1 and 7m3).

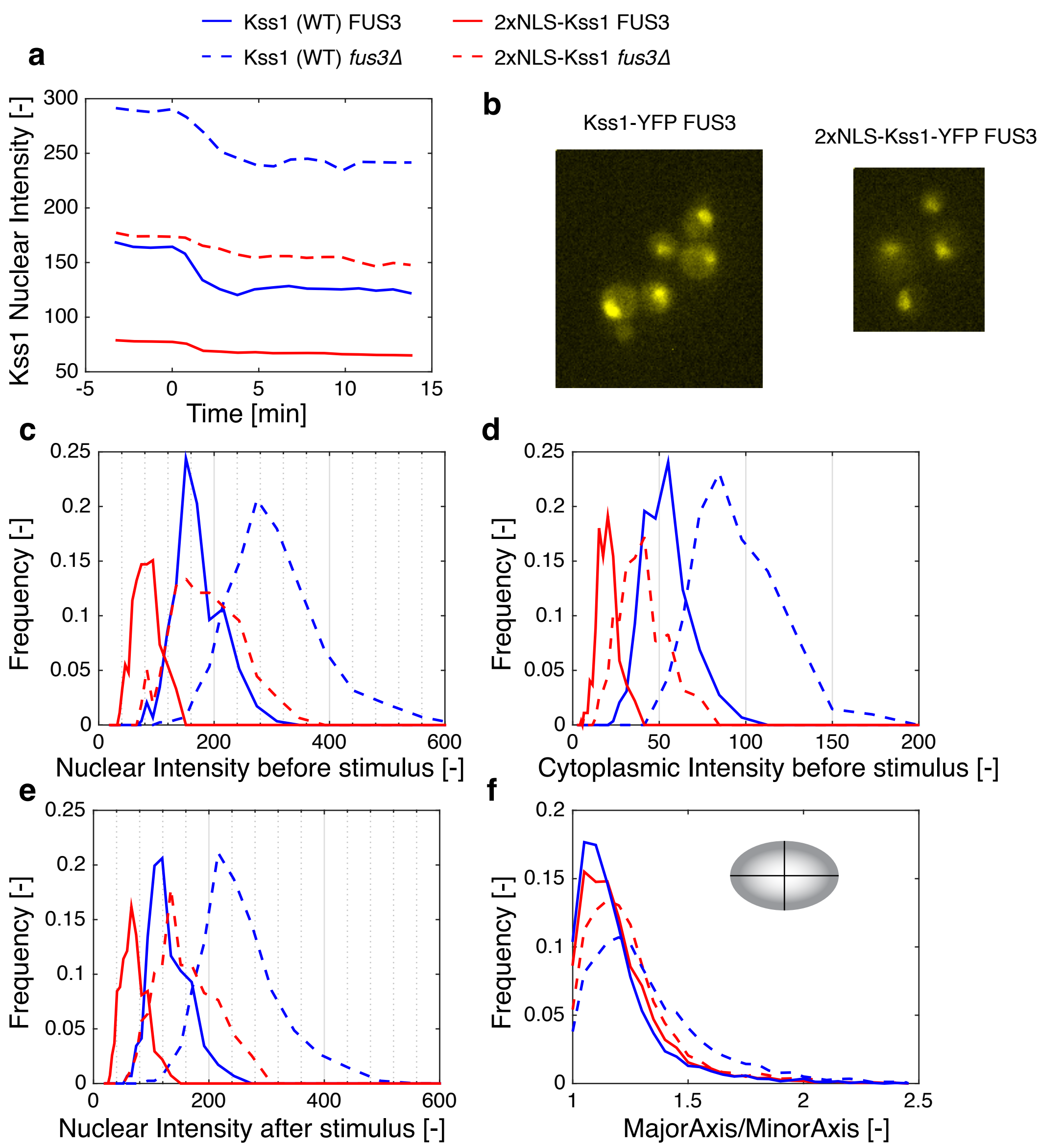

Supplementary Figure 4. Characterization of 2xNLS-Kss1 mutants in WT and *fus3*Δ background
a. Mean nuclear intensity levels of Kss1 and 2xNLS-Kss1 in WT and *fus3*Δ cells as function of time after stimulus. **b.** Kss1-YFP and 2xNLS-Kss1-YFP images from Figures 1B and 4A displayed with the same intensity settings. **c.** and **d.** Histograms of the nuclear (c) and cytoplasmic (d) Kss1 intensity before stimulus **e.** Histograms of the nuclear Kss1 intensity after α-factor treatment . **f.** Ratio of the major axis and minor axis in Kss1 and 2xNLS-Kss1 mutants in WT and *fus3*Δ background of log-phase growing cells. A ratio of 1 denotes a perfect circle, while larger values represent an elliptic morphology.

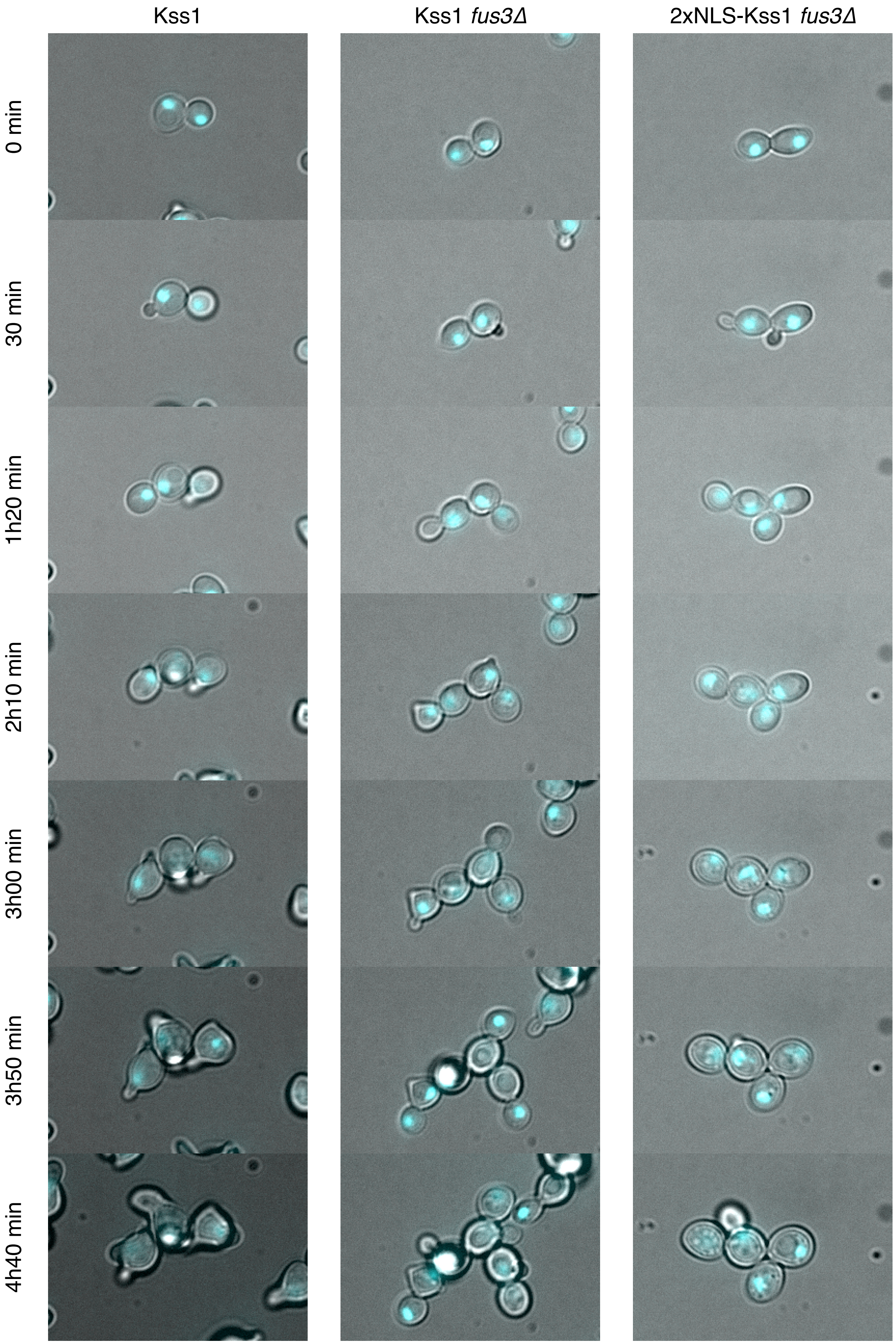

Supplementary Figure 5. Cell cycle arrest in 2xNLS-Kss1 and *fus3*Δ cells.
WT, *fus3*Δ and 2xNLS-Kss1 *fus3*Δ cells were stimulated with alpha-factor 1μM every hour for 5 hours. WT cells shmoo and arrest within an hour after stimulus. *fus3*Δ cells keep dividing and proliferating. 2xNLS-Kss1 *fus3*Δ cells can accomplish a few slow divisions before they arrest as unbudded round cells

Table S1. List of yeast strains used in this study

Strain	Background	Genotype	Plasmid	Figure
ySP2	W303	*MATa leu2-3,112 trp1-1 can1-100 ura3-1 ade2-1 his3-11,15*		
ySP370	W303	*HTA2-CFP*<br>*KSS1-YFP :CaURA3*		1,2,5
ySP385	W303	*HTA2-CFP*<br>*KSS1-YFP :CaURA3*<br>*ste11Δ ::NAT*		2
ySP386	W303	*HTA2-CFP*<br>*KSS1-YFP :CaURA3*<br>*ste7Δ ::NAT*		2
ySP387	W303	*HTA2-CFP*<br>*KSS1-YFP :CaURA3*<br>*ste5Δ ::NAT*		2
ySP388	W303	*HTA2-CFP*<br>*KSS1-YFP :CaURA3*<br>*fus3Δ ::NAT*		2, 5, S4
ySP410	W303	*HTA2-CFP*<br>*KSS1-YFP :CaURA3*<br>*dig1Δ ::NAT*		3
ySP414	W303	*HTA2-CFP*<br>*KSS1-YFP :CaURA3*<br>*ste12Δ ::KAN*		3
ySP415	W303	*HTA2-CFP*<br>*KSS1-YFP :CaURA3*<br>*dig1Δ::NAT*<br>*ste12Δ::KAN*		3
ySP380	W303	*HTA2-CFP*<br>*KSS1-AeF-YFP :CaURA3*	 pSP275	2
ySP409	W303	*HTA2-CFP*<br>*KSS1-AeF-YFP :CaURA3*<br>*fus3Δ ::NAT*	 pSP275	2
ySP451	W303	*HTA2-CFP*<br>*KSS1-AeF-YFP :CaURA3*<br>*fus3Δ ::NAT*<br>*FUS3-as :LEU2*	 pSP275<br><br>pSP198 + pRS305	2
ySP371	W303	*HTA2-CFP*<br>*KSS1-as-YFP :CaURA3*	 pSP274	2
ySP450	W303	*HTA2-CFP*<br>*KSS1-as-YFP :CaURA3*<br>*fus3Δ ::NAT*	 pSP274	2
ySP450	W303	*KSS1-as-YFP :CaURA3*<br>*fus3Δ ::NAT*<br>*pFIG1-qVenus : LEU2*	pSP274<br><br>pSP31	S2
ySP452	W303	*HTA2-CFP*<br>*KSS1-as-YFP :CaURA3*<br>*fus3Δ ::NAT*<br>*FUS3-as :LEU2*	 pSP274<br><br>pSP198 + pRS305	2
ySP432	W303	*HTA2-CFP*<br>*KSS1-D249G-YFP :CaURA3*	 pSP278	3
ySP437	W303	*HTA2-CFP*<br>*KSS1-D249G-YFP :CaURA3*<br>*dig1Δ::NAT*	 pSP278	3
ySP433	W303	*HTA2-CFP*<br>*pADH-KSS1-YFP :CaURA3*	 pSP276	S3
ySP434	W303	*HTA2-CFP*<br>*pADH-KSS1-YFP :CaURA3*<br>*ste12Δ::KAN*	 pSP276	S3
ySP435	W303	*HTA2-CFP*<br>*pADH-KSS1-YFP :CaURA3*<br>*dig1Δ::NAT*	 pSP276	S3
ySP439	W303	*HTA2-CFP*<br>*pADH-KSS1-YFP :CaURA3*<br>*dig1Δ::NAT*<br>*ste12Δ ::KAN*	 pSP276	S3
ySP448	W303	*HTA2-CFP*<br>*KSS1-7m1-YFP :CaURA3*	 pSP279	S3
ySP449	W303	*HTA2-CFP*<br>*KSS1-7m3-YFP :CaURA3*	 pSP280	S3

ySP481	W303	*HTA2-CFP*<br>*2xNLS-KSS1-YFP :CaURA3*	 pSP293	4, 5, S4
ySP482	W303	*HTA2-CFP*<br>*2xNLS-KSS1-YFP :CaURA3*<br>*fus3Δ::NAT*	 pSP293	5, S4
ySP529	W303	*HTA2-CFP*<br>*2xNLS-KSS1-YFP :CaURA3*<br>*far1Δ::NAT*	 pSP293	5
ySP530	W303	*HTA2-CFP*<br>*2xNLS-KSS1-YFP :CaURA3*<br>*far1Δ::NAT*<br>*fus3Δ::KAN*	 pSP293	5
ySP529	W303	*HTA2-CFP*<br>*KSS1-YFP :CaURA3*<br>*far1Δ::NAT*		5
ySP530	W303	*HTA2-CFP*<br>*KSS1-YFP :CaURA3*<br>*far1Δ::NAT*<br>*fus3Δ::KAN*		5
yMS12	W303	*HTA2-CFP :SpHIS5*<br>*pFIG1-qVenus : LEU2*<br>*Kss1-Cherry :CaURA3*	 pSP31<br>pSP313	5, S5
yMS13	W303	*HTA2-CFP :SpHIS5*<br>*pFIG1-qVenus : LEU2*<br>*2xNLS-Kss1-Cherry :CaURA3*	 pSP31<br>pSP316	5
yMS14	W303	*HTA2-CFP :SpHIS5*<br>*pSVS1-qVenus : LEU2*<br>*Kss1-Cherry :CaURA3*	 pSP311<br>pSP313	5
yMS15	W303	*HTA2-CFP :SpHIS5*<br>*pSVS1-qVenus : LEU2*<br>*2xNLS-Kss1-Cherry :CaURA3*	 pSP311<br>pSP316	5
yMS16	W303	*HTA2-CFP :SpHIS5*<br>*pFIG1-qVenus : LEU2*<br>*2xNLS-Kss1-Cherry :CaURA3*<br>*fus3Δ::NAT*	 pSP31<br>pSP316	5, S5
ySP707	W303	*HTA2-CFP :SpHIS5*<br>*pFIG1-qVenus : LEU2*<br>*Kss1-Cherry :CaURA3*<br>*fus3Δ::NAT*	 pSP31<br>pSP313	5, S5
yMS18	W303	*HTA2-CFP :SpHIS5*<br>*pSVS1-qVenus : LEU2*<br>*Kss1-Cherry :CaURA3*<br>*fus3Δ::NAT*	 pSP311<br>pSP313	5
ySP708	W303	*HTA2-CFP :SpHIS5*<br>*pSVS1-qVenus : LEU2*<br>*2xNLS-Kss1-Cherry :CaURA3*<br>*fus3Δ::NAT*	 pSP311<br>pSP316	5
yED205	W303	$Ste7_{DS}$*-SKARS-Cherry:URA3*<br>$Ste7_{ND}$*-SKARS-CFP:LEU2*<br>*Kss1-as-YFP:TRP1*<br>*Hta2-tdiRFP:HIS3*	pED92<br>pED94	1
ySP429	W303	*HTA2-CFP*<br>*FUS3-YFP :CaURA3*		S1

Table S2. List of plasmids used in this study

Plasmid	Insert	Backbone
pSP273	Kss1-WT-mCitrine - CaUra	pRS313
pSP274	Kss1-as-mCitrine - CaUra	pRS313
pSP275	Kss1-(AeF)-mCitrine - CaUra	pRS313
pSP276	pADH-Kss1-mCitrine - CaUra	pRS313
pSP278	Kss1-(D249G))-mCitrine - CaUra	pRS313
pSP279	Kss1-(7m1)-mCitrine - CaUra	pRS313
pSP280	Kss1-(7m3)-mCitrine - CaUra	pRS313
pSP293	2xNLS-Kss1-mCitrine - CaUra	pRS313
pSP313	Kss1-WT-mCherry - CaUra	pRS313
pSP316	2xNLS-Kss1 -mCherry - CaUra	pRS313
pSP198	Fus3-as	pRS406
pSP31	p*FIG1*-qVenus	pRS305
pSP311	p*SVS1*-qVenus	pRS305
pED92	$Ste7_{DS}$-*SKARS-Cherry*	pSIVu
pED94	$Ste7_{ND}$-*SKARS-CFP*	pSIVl
pED101	Kss1-as-mCitrine – synTrp1	pRS313

Table S3. List of supplementary movies

In all movies, time zero corresponds to the time of α-factor addition. In movies 5 to 8, cells were pretreated with NAPP1 5µM or DMSO for 20 to 30 minutes prior to the start of the acquisition. In movie 9, NAPP1 is added before frame 14.

Movie Number	*Strain Imaged*	*Channel Intensity Limits*	*Figure*
Movie 1:	Kss1-YFP wild-type (ySP370)	YFP: 100-400	Figure 1b
Movie 2:	Kss1-YFP *ste5*Δ (ySP387)	YFP: 100-400	Figure 2a
Movie 3:	Kss1-YFP *ste11*Δ (ySP385)	YFP: 100-400	Figure 2a
Movie 4:	Kss1-YFP *fus3*Δ (ySP388)	YFP: 100-900	Figure 2b
Movie 5:	Kss1-AeF-YFP Fus3-as (ySP451) + DMSO	YFP: 100-400	Figure 2d
Movie 6:	Kss1-AeF-YFP Fus3-as (ySP451) + NAPP1	YFP: 100-400	Figure 2d
Movie 7:	Kss1-as-YFP *fus3*Δ (ySP450) + DMSO	YFP: 100-900	Figure 2f
Movie 8:	Kss1-as-YFP *fus3*Δ (ySP450) + NAPP1	YFP: 100-900	Figure 2f
Movie 9:	Kss1-as-YFP Fus3-as (ySP452) + NAPP1	YFP: 100-400	Figure 2g
Movie 10:	Kss1-YFP *dig1*Δ (ySP410)	YFP: 100-750	Figure 3a
Movie 11:	Kss1-YFP *ste12*Δ (ySP414)	YFP: 100-400	Figure 3a
Movie 12:	Kss1-YFP *dig1*Δ*ste12*Δ (ySP415)	YFP: 100-400	Figure 3a
Movie 13:	Kss1-D249G-YFP (ySP432)	YFP: 100-400	Figure 3d
Movie 14:	Kss1-D249G-YFP *dig1*Δ (ySP432)	YFP: 100-1'500	Figure 3d
Movie 15:	2xNLS-Kss1-YFP (ySP481)	YFP: 100-3500	Figure 4a
Movie 16:	Kss1-Cherry, p*FIG1*-qVenus (yMS12)	YFP: 100-10'000<br>RFP 100-1'500	Figure 5a
Movie 17:	2xNLS-Kss1-Cherry, p*FIG1*-qVenus (yMS13)	YFP: 100-10'000<br>RFP 100-1'500	Figure 5a
Movie 18:	Kss1-Cherry, p*FIG1*-qVenus *fus3*Δ (ySP707)	YFP: 100-10'000<br>RFP 100-1'500	Figure 5a
Movie 19:	2xNLS-Kss1-Cherry, p*FIG1*-qVenus *fus3*Δ (yMS16)	YFP: 100-10'000<br>RFP 100-1'500	Figure 5a
